# Development and Validation of Machine Learning Models Incorporating TGFBIp Expression for Prognosis Prediction in Epithelial Ovarian Cancer

**DOI:** 10.1002/cnr2.70745

**Published:** 2026-11-10

**Authors:** Yi Guo, Zhen Li, Wenwei Song, Lansheng Zhang

**Affiliations:** ^1^ Department of Laboratory Medicine The Second Affiliated Hospital of Xuzhou Medical University Xuzhou Jiangsu China; ^2^ Department of Laboratory Medicine The General Hospital of Xuzhou Mining Group Xuzhou Jiangsu China; ^3^ The Second Affiliated Hospital of Xuzhou Medical University Xuzhou Medical University Xuzhou Jiangsu China; ^4^ Department of Oncological Radiotherapy The Second Affiliated Hospital of Xuzhou Medical University Xuzhou Jiangsu China; ^5^ Department of Oncological Radiotherapy The General Hospital of Xuzhou Mining Group Xuzhou Jiangsu China

**Keywords:** immunohistochemistry, machine learning, ovarian cancer, prognostic model, TGFBIp

## Abstract

**Background:**

Epithelial ovarian cancer (EOC) exhibits substantial heterogeneity in clinical outcomes, highlighting the need for diverse prognostic tools. Transforming growth factor‐β‐induced protein (TGFBIp) has been implicated in ovarian cancer progression, but its incremental prognostic value within machine learning frameworks remains unexplored.

**Aims:**

To develop and validate machine learning models incorporating TGFBIp expression to predict vital status and recurrence in EOC patients.

**Methods and Results:**

We analyzed 155 EOC patients from a tissue microarray. TGFBIp expression in tumor cells and stroma was assessed by immunohistochemistry. Twelve machine learning algorithms were benchmarked across four feature inclusion strategies, with TGFBIp expression sequentially added to a baseline clinicopathological model. Model performance was assessed using the area under the receiver operating characteristic curve (AUC) with bootstrap 95% confidence intervals, the area under the precision‐recall curve, calibration intercept and slope, Brier score, and decision curve analysis (DCA). The incremental value of TGFBIp was formally tested by paired bootstrap comparison of test‐set AUCs. SHAP analysis was performed for model interpretation. The support vector machine (SVM) with Strategy 4 (incorporating both tumor and stromal TGFBIp) was selected for vital status prediction, achieving a test‐set AUC of 0.951 (95% CI 0.870–0.998), accuracy of 0.872, sensitivity of 0.920, specificity of 0.818, and negative predictive value (NPV) of 0.900. The random forest (RF) with Strategy 4 was selected for recurrence prediction, achieving a test‐set AUC of 0.936 (95% CI 0.816–1.000), sensitivity of 0.974, and NPV of 0.800. Adding TGFBIp features significantly improved vital status prediction (ΔAUC +0.038, 95% CI +0.002 to +0.092 for Strategy 4 vs. baseline). Calibration was acceptable for the vital status model (test‐set calibration intercept −0.24, slope 1.25, Brier score 0.071). SHAP analysis identified maximum tumor diameter, grade, stage, and TGFBIp expression as leading predictors; tumoral TGFBIp contributed to both outcomes, whereas stromal TGFBIp contributed mainly to vital status prediction. DCA suggested potential clinical utility across a broad range of threshold probabilities.

**Conclusion:**

We established a machine learning framework incorporating compartment‐specific TGFBIp expression for EOC prognosis. TGFBIp in tumor cells provided incremental prognostic information beyond conventional clinicopathological parameters.

## 1 Introduction

Epithelial ovarian cancer (EOC) exhibits substantial heterogeneity in clinical outcomes. With surgery and adjuvant platinum‐based chemotherapy, the 5‐year overall survival of patients with early‐stage disease is 70% to 95% [1, 2]. In contrast, advanced‐stage ovarian cancer, despite treatment with a combination of cytoreductive surgery, platinum‐based chemotherapy, and targeted maintenance therapies, has a 5‐year survival rate of only 10% to 40% [1, 2]. Moreover, even among patients receiving similar treatment regimens, prognosis varies considerably depending on tumor biology, molecular characteristics, and host factors [3]. This wide prognostic disparity underscores the need for diverse risk stratification tools. Therefore, developing additional prognostic models is critical for guiding personalized treatment decisions and improving patient outcomes.

Our previous work demonstrated that platelets promote invasion and induce epithelial–mesenchymal transition (EMT) in ovarian cancer cells through the TGF‐β/Smad signaling pathway [4]. To identify key molecular mediators driving platelet‐induced metastasis, we performed microarray analysis on platelet‐treated ovarian cancer cells and identified transforming growth factor‐β‐induced (TGFBI) as one of the most significantly upregulated genes.

Transforming growth factor‐β‐induced protein (TGFBIp), encoded by the TGFBI gene, interacts with integrins and extracellular matrix components to regulate cell adhesion, migration, and signaling [5]. Accumulating evidence has implicated TGFBIp in cancer progression, though its roles appear context‐dependent across tumor types [6, 7]. In ovarian cancer, we recently demonstrated that TGFBIp promotes migration and invasion by activating the integrin αvβ3‐PI3K‐Akt signaling pathway and inducing EMT [8]. These findings position TGFBIp as a potential biomarker for prognosis prediction.

In recent years, machine learning has emerged as a powerful tool for integrating multidimensional data and capturing complex, non‐linear relationships, offering the potential to improve prognostic accuracy over traditional statistical approaches [9, 10]. Several studies have successfully applied machine learning to predict survival and recurrence in ovarian cancer using clinical, pathological, and genomic features [11, 12]. Nevertheless, the incremental value of incorporating TGFBIp into these models has not been systematically explored.

In this study, we therefore aimed to systematically evaluate the incremental prognostic value of TGFBIp expression for predicting vital status and recurrence in EOC patients. We benchmarked 12 machine learning algorithms across four feature inclusion strategies using a leakage‐free analytical pipeline in which all preprocessing, hyperparameter tuning, and model selection were performed exclusively on the training set, with a held‐out test set reserved for final evaluation. We further employed SHAP analysis to interpret model decisions and decision curve analysis to assess potential clinical utility. To the best of our knowledge, this is the first study to integrate TGFBIp expression into a machine learning prognostic framework for ovarian cancer, thereby expanding the diversity of prognostic tools and offering an additional option for individualized risk stratification research in routine clinical settings.

## 2 Materials and Methods

### 2.1 Tissue Microarray and Immunohistochemical Staining

A human tissue microarray containing ovarian tissues (Cat# HOvaC160Su01, Lot No. T23‐0025) was purchased from Shanghai Outdo Biotech Co. Ltd., together with the corresponding clinicopathological characteristics, follow‐up information, and immunohistochemical staining results of Ki‐67, EGFR, and PD‐L1 for each patient. After excluding five benign cases, 155 patients with EOC were included in the final analysis. IHC staining for TGFBIp was performed on the tissue slides using an immunohistochemical kit (Solarbio, China, Cat# SP0041). Briefly, the slides were deparaffinized, rehydrated in graded ethanol, and subjected to antigen retrieval by microwave heating in sodium citrate buffer. The slides were then incubated overnight at 4°C with a primary antibody against TGFBIp (Proteintech, China, Cat# 10188‐1‐AP) at a dilution of 1:500. After washing with phosphate‐buffered saline, a horseradish peroxidase‐conjugated secondary antibody was added and incubated at 37°C for 30 min. Finally, the sections were visualized with 3,3′‐diaminobenzidine and counterstained with hematoxylin.

TGFBIp expression in tumor cells and stroma was evaluated by multiplying the staining intensity score by the staining proportion. The staining intensity was scored on a 0.25‐step scale (0, 0.25, 0.5, 0.75, 1.0, 1.25, …), and the staining proportion was defined as the percentage of positively stained cells (ranging from 0% to 100%). All sections were independently scored by two pathologists who were blinded to the clinical data, and any discrepancies between the two scorers were resolved by joint review until consensus was reached; the consensus score was used for all analyses. To formally assess inter‐observer reproducibility, the two pathologists independently re‐scored all 155 cores in a blinded manner, without access to the previous scores or clinical data. Inter‐observer agreement was quantified using the two‐way random‐effects, absolute‐agreement intraclass correlation coefficient for single (ICC[2,1]) and average (ICC[2,2]) measurements, with 95% confidence intervals estimated from 2000 bootstrap resamples, together with Bland–Altman analysis.

The available clinicopathological variables comprised age, maximum tumor diameter, histotype, histological grade, FIGO stage, Ki‐67, EGFR, and PD‐L1. The TMA dataset did not contain separate T, N, and M categories; FIGO stage was therefore used as the staging variable, which also avoided redundancy between FIGO stage and TNM categories. Follow‐up information consisted of follow‐up duration and outcome status. Disease recurrence was defined as documented recurrence confirmed according to the Response Evaluation Criteria in Solid Tumors (RECIST). The two study outcomes were vital status (alive or deceased) and disease recurrence (yes or no) at the end of follow‐up. The reporting of this study adheres to the TRIPOD+AI guideline where applicable [13].

### 2.2 Study Design and Data Preprocessing

To prevent data leakage, the dataset was first partitioned into a training set (70%, *n* = 108) and a held‐out test set (30%, *n* = 47) using stratified sampling based on the outcome variable to preserve class distribution (random seed 42). All subsequent preprocessing steps were fitted on the training set only and then applied to the test set: missing values in continuous variables were imputed using the median of the respective feature computed from the training set; categorical variables (histotype, grade, FIGO stage) were transformed by one‐hot encoding; and all continuous features were standardized using z‐score normalization with the means and standard deviations estimated from the training set. The baseline feature set (Strategy 1) comprised age, maximum tumor diameter, Ki‐67, EGFR, PD‐L1, histotype, grade, and FIGO stage. Three additional feature inclusion strategies were defined to evaluate the incremental value of TGFBIp: Strategy 2 added TGFBIp expression in tumor cells, Strategy 3 added TGFBIp expression in stroma, and Strategy 4 added both. Multicollinearity was assessed using the variance inflation factor (VIF) on the one‐hot encoded Strategy 4 design matrix of the training set; all VIFs were below 12 (Table S2).

Continuous variables were presented as median (interquartile range) and were compared between groups using the Mann–Whitney U test; categorical variables were compared using the chi‐square test or Fisher's exact test, as appropriate. All statistical tests were two‐sided, and a *p* value < 0.05 was considered statistically significant.

### 2.3 Machine Learning Model Development and Selection

Twelve machine learning algorithms were benchmarked: Naive Bayes (NB), logistic regression (LR), support vector machine (SVM), K‐nearest neighbors (KNN), artificial neural network (ANN), decision tree (DT), gradient boosting decision tree (GBDT), random forest (RF), light gradient boosting machine (LightGBM), extreme gradient boosting (XGBoost), categorical boosting (CatBoost), and adaptive boosting (AdaBoost). All models were trained to predict two binary outcomes: vital status and disease recurrence.

For each combination of algorithm and feature strategy (48 combinations per outcome), hyperparameters were optimized by grid search with stratified 10‐fold cross‐validation on the training set only (Table S1). The final algorithm and feature strategy were then selected exclusively on the training set using repeated stratified 5‐fold cross‐validation with 10 repeats (50 performance estimates per combination), in a two‐step procedure: first, the algorithm with the highest mean repeated cross‐validation AUC was identified; second, feature strategies were compared within the selected algorithm, applying the one‐standard‐error rule and using the out‐of‐fold calibration slope as a tie‐breaker. The held‐out test set was not used for model selection in any form and was evaluated exactly once, after the final model had been fixed. This study is therefore a development study with internal validation (a held‐out test set from the same cohort), not external validation.

### 2.4 Model Evaluation

Model performance was quantified using the AUC with 95% confidence intervals estimated by bootstrap resampling (2000 resamples), accuracy, sensitivity, specificity, positive predictive value (PPV), negative predictive value (NPV), and F1‐score at the default probability threshold of 0.5, together with the area under the precision‐recall curve (AUPRC), which is informative under class imbalance. Calibration was assessed with calibration curves (10 quantile‐based bins), the calibration intercept and calibration slope estimated by logistic recalibration, and the Brier score. The incremental prognostic value of TGFBIp was formally tested by comparing the test‐set AUCs of Strategies 2–4 against Strategy 1 using paired bootstrap resampling (2000 resamples); a 95% confidence interval for ΔAUC excluding zero was interpreted as statistically significant incremental value. As a sensitivity analysis, the incremental value comparison was repeated using LR as a reference algorithm. To assess robustness to class imbalance, the final models were additionally evaluated with class‐weighted training.

### 2.5 Decision Curve Analysis

DCA was performed to evaluate the potential clinical net benefit of the selected models. Net benefit was calculated as NB = TP/*N* − FP/*N* × [*t*/(1 − *t*)], where TP and FP represent true and false positives, *N* is the total sample size, and *t* is the threshold probability. The models were compared against “treat all” and “treat none” reference strategies across threshold probabilities from 0.01 to 0.99.

### 2.6 SHAP‐Based Model Interpretation

To enhance the interpretability of the final models, we performed SHapley Additive exPlanations (SHAP) analysis [14]. For the SVM model, SHAP values were computed using the model‐agnostic KernelExplainer with a k‐means‐summarized background dataset (25 clusters) and 200 perturbation samples per evaluation; for the RF model, the exact TreeExplainer was used. SHAP summary plots were generated to visualize the distribution of SHAP values, with features ranked by their mean absolute SHAP values; the top 10 features were displayed. All analyses were conducted in Python 3 using scikit‐learn and the SHAP library.

## 3 Results

### 3.1 Baseline Characteristics of the Study Population

A total of 155 EOC patients from the tissue microarray were included in the final analysis. The median age was 52.0 years (IQR 44.0–60.0). During follow‐up (median overall follow‐up 64 months, IQR 30–83, range 4–109), 125 patients (80.6%) experienced recurrence and 83 patients (53.5%) died. After stratified splitting, the training set (*n* = 108) comprised 58 deceased and 50 alive patients and 87 recurrent and 21 non‐recurrent patients, and the test set (*n* = 47) comprised 25 deceased and 22 alive patients and 38 recurrent and 9 non‐recurrent patients. The proportions of missing values were 10.3% for Ki‐67 (16 values), 8.4% for EGFR (13 values), 5.2% for PD‐L1 (8 values), and 0.6% for age (1 value); missing values were handled by median imputation within the training set. Representative immunohistochemical staining of TGFBIp at graded intensities is shown in Figure 1.

**FIGURE 1:**
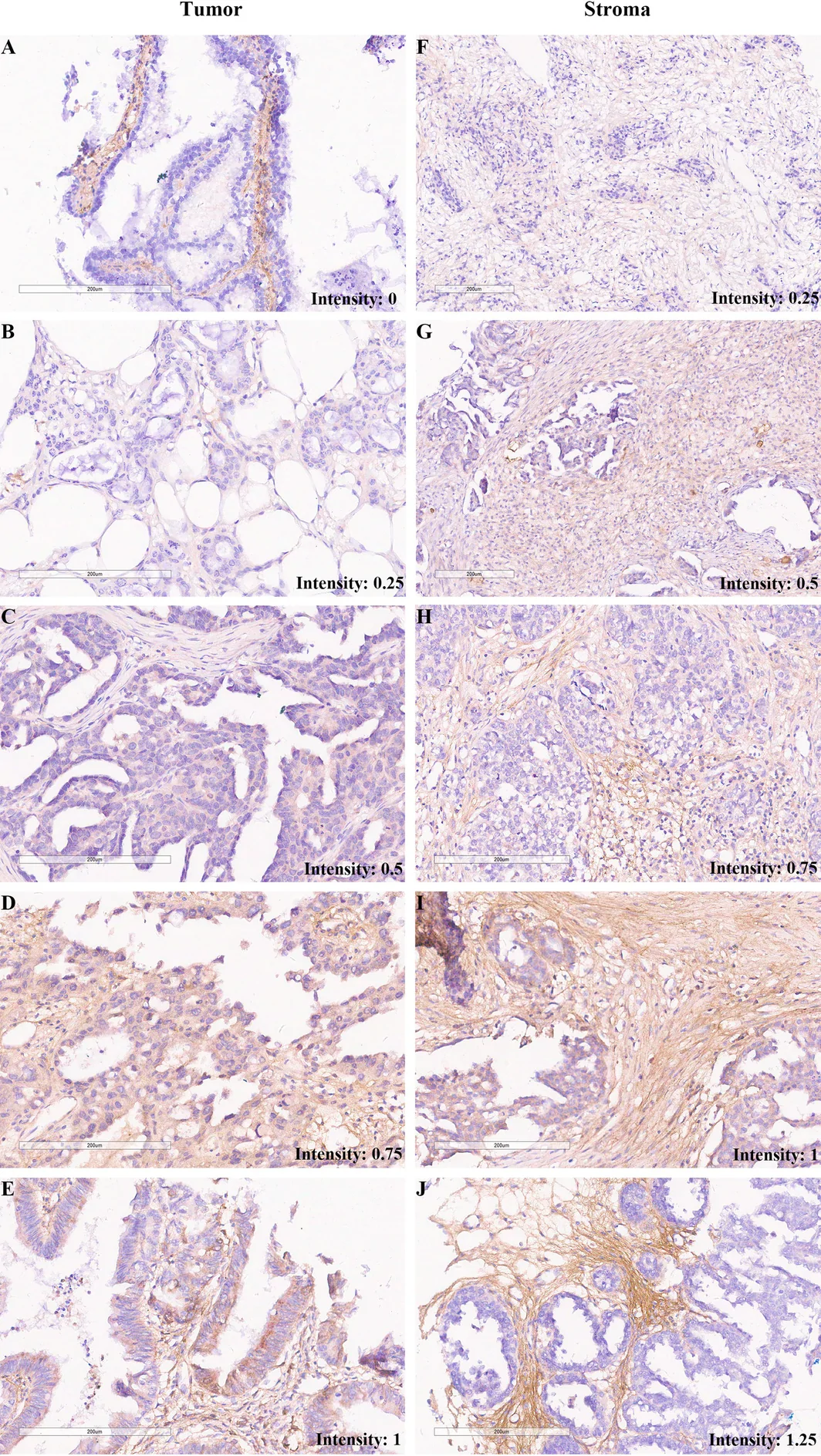
Representative immunohistochemical staining of TGFBIp expression intensity in ovarian cancer tissues. (A–E) Representative images showing graded TGFBIp expression intensity in tumor cells, with intensity scores of 0, 0.25, 0.50, 0.75, and 1.00, respectively. (F–J) Representative images showing graded TGFBIp expression intensity in tumor stroma, with intensity scores of 0.25, 0.50, 0.75, 1.00, and 1.25, respectively. Scale bar = 200 μm. TGFBIp, transforming growth factor‐β‐induced protein.

Table 1 summarizes the characteristics of patients stratified by vital status. Compared with alive patients, deceased patients were significantly older (median 54.0 vs. 48.0 years, *p* = 0.010), had larger tumors (median maximum diameter 15.3 vs. 8.0 cm, *p* < 0.001), and were more likely to have serous carcinoma (*p* = 0.027), Grade III tumors (*p* < 0.001), and Stage III–IV disease (*p* < 0.001). Importantly, TGFBIp expression was significantly higher in deceased patients, both in tumor cells (median 50.0 vs. 12.5, *p* < 0.001) and in stroma (median 75.0 vs. 50.0, *p* < 0.001), whereas Ki‐67, EGFR, and PD‐L1 did not differ significantly between the groups.

**TABLE 1:** Baseline characteristics of the 155 patients by vital status.

Characteristic	Alive (*n* = 72)	Deceased (*n* = 83)	*p*
Age, years	48.00 (41.75, 56.00)	54.00 (46.00, 62.00)	0.010
Maximum tumor diameter, cm	7.95 (4.60, 13.07)	15.30 (11.95, 17.40)	< 0.001
ki67, %	35.00 (15.00, 67.50)	52.50 (10.62, 89.38)	0.093
EGFR score	30.00 (5.00, 80.00)	25.00 (5.00, 70.00)	0.754
PDL1 score	0.50 (0.00, 2.50)	0.50 (0.00, 3.75)	0.187
TGFBIp expression in tumor	12.50 (0.00, 25.00)	50.00 (25.00, 67.50)	< 0.001
TGFBIp expression in stroma	50.00 (50.00, 75.00)	75.00 (56.25, 100.00)	< 0.001
Histotype			0.027
Clear cell carcinoma	1 (1.4%)	2 (2.4%)	
Endometrioid carcinoma	10 (13.9%)	6 (7.2%)	
Mucinous carcinoma	22 (30.6%)	12 (14.5%)	
Serous carcinoma	39 (54.2%)	63 (75.9%)	
Grade			< 0.001
I	30 (41.7%)	6 (7.2%)	
II	20 (27.8%)	3 (3.6%)	
III	22 (30.6%)	74 (89.2%)	
FIGO stage			< 0.001
I	9 (12.5%)	0 (0.0%)	
II	32 (44.4%)	4 (4.8%)	
III	31 (43.1%)	46 (55.4%)	
IV	0 (0.0%)	33 (39.8%)	

*Note:* Continuous variables are summarized as median (interquartile range) and were compared with the Mann–Whitney U test; categorical variables are summarized as *n* (%) and were compared with the chi‐square test.

Abbreviation: TGFBIp, transforming growth factor‐β‐induced protein.

As shown in Table 2, patients with recurrence were significantly older (*p* = 0.001), had larger tumors (*p* = 0.041), higher‐grade tumors (*p* < 0.001), and more advanced FIGO stage (*p* < 0.001) than non‐recurrent patients. TGFBIp expression in tumor cells was significantly higher in recurrent patients (median 30.0 vs. 5.0, *p* < 0.001), and stromal TGFBIp showed a similar trend (*p* = 0.071). Histotype distribution, Ki‐67, EGFR, and PD‐L1 did not differ significantly between the two groups.

**TABLE 2:** Baseline characteristics of the 155 patients by recurrence status.

Characteristic	No recurrence (*n* = 30)	Recurrence (*n* = 125)	*p*
Age, years	44.50 (41.25, 51.75)	53.50 (45.00, 61.00)	0.001
Maximum tumor diameter, cm	8.35 (4.72, 15.05)	13.20 (8.00, 16.90)	0.041
ki67, %	32.50 (12.19, 69.38)	50.00 (12.50, 80.00)	0.350
EGFR score	26.25 (5.00, 58.12)	27.50 (5.62, 70.00)	0.644
PDL1 score	0.25 (0.00, 2.50)	0.50 (0.00, 5.00)	0.148
TGFBIp expression in tumor	5.00 (0.00, 12.50)	30.00 (25.00, 50.00)	< 0.001
TGFBIp expression in stroma	50.00 (50.00, 75.00)	75.00 (50.00, 100.00)	0.071
Histotype			0.454
Clear cell carcinoma	0 (0.0%)	3 (2.4%)	
Endometrioid carcinoma	4 (13.3%)	12 (9.6%)	
Mucinous carcinoma	9 (30.0%)	25 (20.0%)	
Serous carcinoma	17 (56.7%)	85 (68.0%)	
Grade			< 0.001
I	14 (46.7%)	22 (17.6%)	
II	11 (36.7%)	12 (9.6%)	
III	5 (16.7%)	91 (72.8%)	
FIGO stage			< 0.001
I	8 (26.7%)	1 (0.8%)	
II	20 (66.7%)	16 (12.8%)	
III	2 (6.7%)	75 (60.0%)	
IV	0 (0.0%)	33 (26.4%)	

*Note:* Continuous variables are summarized as median (interquartile range) and were compared with the Mann–Whitney U test; categorical variables are summarized as *n* (%) and were compared with the chi‐square test.

Abbreviation: TGFBIp, transforming growth factor‐β‐induced protein.

Taken together, conventional clinicopathological parameters and TGFBIp expression—particularly tumoral TGFBIp—were associated with both outcomes in unadjusted group comparisons, supporting the hypothesis that TGFBIp carries prognostic information. To formally quantify its incremental value within a predictive modeling framework, four feature inclusion strategies were compared as described in Section 2.

Inter‐observer agreement for TGFBIp scoring, assessed in the blinded independent re‐scoring of all 155 cores, was moderate to good for the continuous scores: ICC(2,1) was 0.680 (95% CI 0.550–0.788) for tumoral TGFBIp and 0.682 (0.544–0.795) for stromal TGFBIp, and the average‐measures ICC(2,2)—reflecting the reliability of the two‐rater consensus process used in this study—was 0.810 (0.709–0.882) and 0.811 (0.704–0.886), respectively. Bland–Altman analysis showed negligible mean bias between raters (1.5 points for tumor and 1.4 points for stroma; Figure S3). Exact agreement on staining intensity was 69.0% for tumor and 85.2% for stroma, and agreement within one intensity step was 76.8% and 87.7%, respectively.

### 3.2 Model Selection on the Training Set

Model selection was performed exclusively on the training set using repeated stratified 5‐fold cross‐validation (10 repeats). For vital status, SVM with Strategy 4 achieved the highest mean repeated cross‐validation AUC (0.9375 ± 0.0413; Table S3), followed by XGBoost with Strategy 4 (0.9360 ± 0.0474) and ANN with Strategy 4 (0.9358 ± 0.0458); SVM with Strategy 4 also showed the most favorable out‐of‐fold calibration among the leading candidates (calibration slope 1.11) and was therefore selected as the final vital status model. For recurrence, RF with Strategy 4 ranked first (0.9236 ± 0.0618; Table S4), with RF with Strategy 2 falling within one standard error (0.9212 ± 0.0633); Strategy 4 was retained because it captures the complementary contribution of both TGFBIp compartments, which is central to the study hypothesis. Notably, the prespecified selection procedure identified Strategy 4—the strategy incorporating both tumor and stromal TGFBIp—as the winning feature strategy for both outcomes.

### 3.3 Performance of the Final Models

The performance of all 48 candidate models for each outcome is reported in Tables 3, 4, 5, 6. The final models were evaluated once on the held‐out test set.

**TABLE 3:** Training‐set performance of the 48 candidate models for vital status prediction.

Feature strategy	Algorithm	AUC	Accuracy	Sensitivity	Specificity	PPV	NPV	F1
Strategy 1	NB	0.942	0.806	0.983	0.600	0.740	0.968	0.844
	LR	0.969	0.917	0.914	0.920	0.930	0.902	0.922
	SVM	0.971	0.926	0.948	0.900	0.917	0.938	0.932
	KNN	1.000	1.000	1.000	1.000	1.000	1.000	1.000
	ANN	1.000	0.991	0.983	1.000	1.000	0.980	0.991
	DT	0.970	0.889	0.931	0.840	0.871	0.913	0.900
	GBDT	1.000	1.000	1.000	1.000	1.000	1.000	1.000
	RF	0.980	0.917	0.966	0.860	0.889	0.956	0.926
	LightGBM	0.988	0.926	0.914	0.940	0.946	0.904	0.930
	XGBoost	0.999	0.991	0.983	1.000	1.000	0.980	0.991
	CatBoost	0.980	0.917	0.914	0.920	0.930	0.902	0.922
	AdaBoost	0.974	0.889	0.914	0.860	0.883	0.896	0.898
Strategy 2	NB	0.945	0.806	0.983	0.600	0.740	0.968	0.844
	LR	0.973	0.926	0.914	0.940	0.946	0.904	0.930
	SVM	0.964	0.898	0.862	0.940	0.943	0.855	0.901
	KNN	0.938	0.815	0.931	0.680	0.771	0.895	0.844
	ANN	1.000	0.991	1.000	0.980	0.983	1.000	0.991
	DT	0.916	0.870	0.897	0.840	0.867	0.875	0.881
	GBDT	1.000	1.000	1.000	1.000	1.000	1.000	1.000
	RF	0.984	0.944	0.966	0.920	0.933	0.958	0.949
	LightGBM	0.999	0.981	0.983	0.980	0.983	0.980	0.983
	XGBoost	1.000	1.000	1.000	1.000	1.000	1.000	1.000
	CatBoost	1.000	1.000	1.000	1.000	1.000	1.000	1.000
	AdaBoost	1.000	1.000	1.000	1.000	1.000	1.000	1.000
Strategy 3	NB	0.942	0.806	0.983	0.600	0.740	0.968	0.844
	LR	0.966	0.917	0.914	0.920	0.930	0.902	0.922
	SVM	0.939	0.880	0.862	0.900	0.909	0.849	0.885
	KNN	1.000	1.000	1.000	1.000	1.000	1.000	1.000
	ANN	1.000	1.000	1.000	1.000	1.000	1.000	1.000
	DT	0.916	0.870	0.897	0.840	0.867	0.875	0.881
	GBDT	1.000	1.000	1.000	1.000	1.000	1.000	1.000
	RF	0.982	0.944	0.948	0.940	0.948	0.940	0.948
	LightGBM	0.986	0.926	0.931	0.920	0.931	0.920	0.931
	XGBoost	0.988	0.954	0.966	0.940	0.949	0.959	0.957
	CatBoost	0.986	0.944	0.948	0.940	0.948	0.940	0.948
	AdaBoost	0.973	0.889	0.914	0.860	0.883	0.896	0.898
Strategy 4	NB	0.946	0.806	0.983	0.600	0.740	0.968	0.844
	LR	0.976	0.935	0.931	0.940	0.947	0.922	0.939
	SVM	0.974	0.926	0.948	0.900	0.917	0.938	0.932
	KNN	1.000	1.000	1.000	1.000	1.000	1.000	1.000
	ANN	1.000	0.991	0.983	1.000	1.000	0.980	0.991
	DT	0.970	0.889	0.931	0.840	0.871	0.913	0.900
	GBDT	1.000	1.000	1.000	1.000	1.000	1.000	1.000
	RF	0.986	0.954	0.966	0.940	0.949	0.959	0.957
	LightGBM	0.987	0.935	0.931	0.940	0.947	0.922	0.939
	XGBoost	0.990	0.954	0.948	0.960	0.965	0.941	0.957
	CatBoost	0.989	0.963	0.966	0.960	0.966	0.960	0.966
	AdaBoost	0.986	0.935	0.966	0.900	0.918	0.957	0.941

*Note:* Strategy 1, baseline clinicopathological features (age, maximum tumor diameter, ki67, EGFR, PD‐L1, histotype, grade, FIGO stage); Strategy 2, Strategy 1 + tumoral TGFBIp; Strategy 3, Strategy 1 + stromal TGFBIp; Strategy 4, Strategy 1 + both. For the test set, the AUC is given with the bootstrap 95% confidence interval (2000 resamples). Classification metrics use the 0.5 probability threshold.

Abbreviations: NPV, negative predictive value; PPV, positive predictive value.

**TABLE 4:** Held‐out test‐set performance of the 48 candidate models for vital status prediction.

Feature strategy	Algorithm	AUC	Accuracy	Sensitivity	Specificity	PPV	NPV	F1
Strategy 1	NB	0.860 (0.724–0.969)	0.723	0.880	0.545	0.688	0.800	0.772
	LR	0.920 (0.824–0.987)	0.851	0.880	0.818	0.846	0.857	0.863
	SVM	0.915 (0.817–0.985)	0.851	0.880	0.818	0.846	0.857	0.863
	KNN	0.875 (0.765–0.969)	0.787	0.880	0.682	0.759	0.833	0.815
	ANN	0.867 (0.737–0.965)	0.851	0.880	0.818	0.846	0.857	0.863
	DT	0.797 (0.649–0.912)	0.723	0.840	0.591	0.700	0.765	0.764
	GBDT	0.882 (0.780–0.967)	0.809	0.800	0.818	0.833	0.783	0.816
	RF	0.925 (0.837–0.989)	0.872	0.920	0.818	0.852	0.900	0.885
	LightGBM	0.891 (0.777–0.974)	0.809	0.800	0.818	0.833	0.783	0.816
	XGBoost	0.896 (0.793–0.973)	0.830	0.840	0.818	0.840	0.818	0.840
	CatBoost	0.909 (0.806–0.982)	0.872	0.920	0.818	0.852	0.900	0.885
	AdaBoost	0.918 (0.833–0.984)	0.872	0.960	0.773	0.828	0.944	0.889
Strategy 2	NB	0.871 (0.742–0.978)	0.723	0.880	0.545	0.688	0.800	0.772
	LR	0.955 (0.885–1.000)	0.936	0.960	0.909	0.923	0.952	0.941
	SVM	0.929 (0.830–0.998)	0.872	0.800	0.955	0.952	0.808	0.870
	KNN	0.928 (0.830–1.000)	0.851	0.960	0.727	0.800	0.941	0.873
	ANN	0.918 (0.824–0.985)	0.851	0.920	0.773	0.821	0.895	0.868
	DT	0.816 (0.693–0.919)	0.723	0.800	0.636	0.714	0.737	0.755
	GBDT	0.931 (0.851–0.991)	0.851	0.840	0.864	0.875	0.826	0.857
	RF	0.951 (0.869–1.000)	0.936	0.960	0.909	0.923	0.952	0.941
	LightGBM	0.922 (0.824–0.989)	0.894	0.880	0.909	0.917	0.870	0.898
	XGBoost	0.948 (0.870–0.996)	0.872	0.880	0.864	0.880	0.864	0.880
	CatBoost	0.944 (0.864–0.996)	0.894	0.880	0.909	0.917	0.870	0.898
	AdaBoost	0.922 (0.828–0.987)	0.894	0.920	0.864	0.885	0.905	0.902
Strategy 3	NB	0.867 (0.735–0.974)	0.723	0.880	0.545	0.688	0.800	0.772
	LR	0.944 (0.873–0.994)	0.894	0.960	0.818	0.857	0.947	0.906
	SVM	0.905 (0.806–0.985)	0.894	0.920	0.864	0.885	0.905	0.902
	KNN	0.871 (0.752–0.966)	0.766	0.760	0.773	0.792	0.739	0.776
	ANN	0.915 (0.812–0.989)	0.894	0.960	0.818	0.857	0.947	0.906
	DT	0.816 (0.693–0.919)	0.723	0.800	0.636	0.714	0.737	0.755
	GBDT	0.867 (0.748–0.951)	0.809	0.880	0.727	0.786	0.842	0.830
	RF	0.929 (0.833–0.991)	0.872	0.920	0.818	0.852	0.900	0.885
	LightGBM	0.898 (0.795–0.973)	0.809	0.800	0.818	0.833	0.783	0.816
	XGBoost	0.918 (0.825–0.985)	0.809	0.880	0.727	0.786	0.842	0.830
	CatBoost	0.911 (0.812–0.982)	0.894	0.920	0.864	0.885	0.905	0.902
	AdaBoost	0.916 (0.831–0.983)	0.872	0.960	0.773	0.828	0.944	0.889
Strategy 4	NB	0.873 (0.745–0.979)	0.723	0.880	0.545	0.688	0.800	0.772
	LR	0.956 (0.891–0.998)	0.915	0.960	0.864	0.889	0.950	0.923
	SVM	0.951 (0.870–0.998)	0.872	0.920	0.818	0.852	0.900	0.885
	KNN	0.917 (0.831–0.987)	0.851	0.880	0.818	0.846	0.857	0.863
	ANN	0.955 (0.875–1.000)	0.915	0.960	0.864	0.889	0.950	0.923
	DT	0.797 (0.649–0.912)	0.723	0.840	0.591	0.700	0.765	0.764
	GBDT	0.945 (0.870–1.000)	0.872	0.920	0.818	0.852	0.900	0.885
	RF	0.949 (0.862–1.000)	0.936	0.960	0.909	0.923	0.952	0.941
	LightGBM	0.955 (0.879–1.000)	0.894	0.880	0.909	0.917	0.870	0.898
	XGBoost	0.945 (0.862–1.000)	0.915	0.960	0.864	0.889	0.950	0.923
	CatBoost	0.951 (0.869–1.000)	0.915	0.960	0.864	0.889	0.950	0.923
	AdaBoost	0.965 (0.899–1.000)	0.936	0.960	0.909	0.923	0.952	0.941

*Note:* Strategy 1, baseline clinicopathological features (age, maximum tumor diameter, ki67, EGFR, PD‐L1, histotype, grade, FIGO stage); Strategy 2, Strategy 1 + tumoral TGFBIp; Strategy 3, Strategy 1 + stromal TGFBIp; Strategy 4, Strategy 1 + both. For the test set, the AUC is given with the bootstrap 95% confidence interval (2000 resamples). Classification metrics use the 0.5 probability threshold.

Abbreviations: NPV, negative predictive value; PPV, positive predictive value.

**TABLE 5:** Training‐set performance of the 48 candidate models for recurrence prediction.

Feature strategy	Algorithm	AUC	Accuracy	Sensitivity	Specificity	PPV	NPV	F1
Strategy 1	NB	0.956	0.778	0.724	1.000	1.000	0.467	0.840
	LR	0.958	0.898	0.931	0.762	0.942	0.727	0.936
	SVM	0.959	0.898	0.943	0.714	0.932	0.750	0.937
	KNN	1.000	1.000	1.000	1.000	1.000	1.000	1.000
	ANN	1.000	1.000	1.000	1.000	1.000	1.000	1.000
	DT	0.918	0.861	0.874	0.810	0.950	0.607	0.910
	GBDT	0.999	0.981	1.000	0.905	0.978	1.000	0.989
	RF	1.000	0.991	1.000	0.952	0.989	1.000	0.994
	LightGBM	1.000	0.991	1.000	0.952	0.989	1.000	0.994
	XGBoost	1.000	0.972	1.000	0.857	0.967	1.000	0.983
	CatBoost	0.993	0.954	1.000	0.762	0.946	1.000	0.972
	AdaBoost	0.993	0.972	1.000	0.857	0.967	1.000	0.983
Strategy 2	NB	0.957	0.769	0.713	1.000	1.000	0.457	0.832
	LR	0.941	0.843	0.977	0.286	0.850	0.750	0.909
	SVM	0.966	0.907	0.954	0.714	0.933	0.789	0.943
	KNN	1.000	1.000	1.000	1.000	1.000	1.000	1.000
	ANN	1.000	0.991	1.000	0.952	0.989	1.000	0.994
	DT	0.963	0.926	0.920	0.952	0.988	0.741	0.952
	GBDT	1.000	1.000	1.000	1.000	1.000	1.000	1.000
	RF	1.000	0.991	1.000	0.952	0.989	1.000	0.994
	LightGBM	0.997	0.954	0.977	0.857	0.966	0.900	0.971
	XGBoost	1.000	1.000	1.000	1.000	1.000	1.000	1.000
	CatBoost	1.000	1.000	1.000	1.000	1.000	1.000	1.000
	AdaBoost	0.994	0.981	1.000	0.905	0.978	1.000	0.989
Strategy 3	NB	0.956	0.778	0.724	1.000	1.000	0.467	0.840
	LR	0.958	0.898	0.931	0.762	0.942	0.727	0.936
	SVM	0.967	0.880	0.966	0.524	0.894	0.786	0.928
	KNN	1.000	1.000	1.000	1.000	1.000	1.000	1.000
	ANN	1.000	1.000	1.000	1.000	1.000	1.000	1.000
	DT	0.918	0.861	0.874	0.810	0.950	0.607	0.910
	GBDT	1.000	1.000	1.000	1.000	1.000	1.000	1.000
	RF	0.989	0.954	1.000	0.762	0.946	1.000	0.972
	LightGBM	0.991	0.963	1.000	0.810	0.956	1.000	0.978
	XGBoost	0.986	0.963	1.000	0.810	0.956	1.000	0.978
	CatBoost	1.000	1.000	1.000	1.000	1.000	1.000	1.000
	AdaBoost	0.992	0.972	1.000	0.857	0.967	1.000	0.983
Strategy 4	NB	0.957	0.759	0.701	1.000	1.000	0.447	0.824
	LR	0.962	0.917	0.966	0.714	0.933	0.833	0.949
	SVM	0.969	0.907	0.966	0.667	0.923	0.824	0.944
	KNN	1.000	1.000	1.000	1.000	1.000	1.000	1.000
	ANN	1.000	1.000	1.000	1.000	1.000	1.000	1.000
	DT	0.994	0.972	0.989	0.905	0.977	0.950	0.983
	GBDT	0.999	0.991	1.000	0.952	0.989	1.000	0.994
	RF	0.993	0.935	1.000	0.667	0.926	1.000	0.961
	LightGBM	0.996	0.954	0.989	0.810	0.956	0.944	0.972
	XGBoost	0.998	0.981	1.000	0.905	0.978	1.000	0.989
	CatBoost	1.000	1.000	1.000	1.000	1.000	1.000	1.000
	AdaBoost	0.988	0.954	0.977	0.857	0.966	0.900	0.971

*Note:* Strategy 1, baseline clinicopathological features (age, maximum tumor diameter, ki67, EGFR, PD‐L1, histotype, grade, FIGO stage); Strategy 2, Strategy 1 + tumoral TGFBIp; Strategy 3, Strategy 1 + stromal TGFBIp; Strategy 4, Strategy 1 + both. For the test set, the AUC is given with the bootstrap 95% confidence interval (2000 resamples). Classification metrics use the 0.5 probability threshold.

Abbreviations: NPV, negative predictive value; PPV, positive predictive value.

**TABLE 6:** Held‐out test‐set performance of the 48 candidate models for recurrence prediction.

Feature strategy	Algorithm	AUC	Accuracy	Sensitivity	Specificity	PPV	NPV	F1
Strategy 1	NB	0.880 (0.685–0.995)	0.745	0.711	0.889	0.964	0.421	0.818
	LR	0.865 (0.699–0.984)	0.872	0.947	0.556	0.900	0.714	0.923
	SVM	0.822 (0.643–0.974)	0.894	0.974	0.556	0.902	0.833	0.937
	KNN	0.857 (0.710–0.971)	0.894	1.000	0.444	0.884	1.000	0.938
	ANN	0.749 (0.492–0.976)	0.872	0.947	0.556	0.900	0.714	0.923
	DT	0.800 (0.610–0.963)	0.851	0.868	0.778	0.943	0.583	0.904
	GBDT	0.904 (0.776–0.992)	0.872	0.947	0.556	0.900	0.714	0.923
	RF	0.915 (0.795–0.992)	0.872	0.974	0.444	0.881	0.800	0.925
	LightGBM	0.763 (0.532–0.954)	0.851	0.974	0.333	0.860	0.750	0.914
	XGBoost	0.835 (0.602–0.983)	0.872	0.974	0.444	0.881	0.800	0.925
	CatBoost	0.915 (0.804–0.990)	0.872	0.974	0.444	0.881	0.800	0.925
	AdaBoost	0.865 (0.667–0.987)	0.851	0.974	0.333	0.860	0.750	0.914
Strategy 2	NB	0.892 (0.689–1.000)	0.745	0.711	0.889	0.964	0.421	0.818
	LR	0.892 (0.760–0.987)	0.894	1.000	0.444	0.884	1.000	0.938
	SVM	0.912 (0.797–0.988)	0.894	0.974	0.556	0.902	0.833	0.937
	KNN	0.870 (0.703–0.986)	0.894	0.974	0.556	0.902	0.833	0.937
	ANN	0.827 (0.612–0.978)	0.872	0.947	0.556	0.900	0.714	0.923
	DT	0.807 (0.635–0.961)	0.851	0.868	0.778	0.943	0.583	0.904
	GBDT	0.915 (0.776–1.000)	0.915	0.947	0.778	0.947	0.778	0.947
	RF	0.942 (0.833–1.000)	0.894	0.974	0.556	0.902	0.833	0.937
	LightGBM	0.921 (0.757–1.000)	0.894	0.974	0.556	0.902	0.833	0.937
	XGBoost	0.927 (0.773–1.000)	0.915	0.974	0.667	0.925	0.857	0.949
	CatBoost	0.953 (0.867–1.000)	0.915	0.974	0.667	0.925	0.857	0.949
	AdaBoost	0.915 (0.794–1.000)	0.894	0.947	0.667	0.923	0.750	0.935
Strategy 3	NB	0.886 (0.688–1.000)	0.745	0.711	0.889	0.964	0.421	0.818
	LR	0.865 (0.699–0.984)	0.872	0.947	0.556	0.900	0.714	0.923
	SVM	0.868 (0.712–0.980)	0.872	0.974	0.444	0.881	0.800	0.925
	KNN	0.711 (0.492–0.918)	0.894	0.974	0.556	0.902	0.833	0.937
	ANN	0.743 (0.492–0.967)	0.872	0.947	0.556	0.900	0.714	0.923
	DT	0.800 (0.610–0.963)	0.851	0.868	0.778	0.943	0.583	0.904
	GBDT	0.906 (0.785–0.990)	0.894	0.947	0.667	0.923	0.750	0.935
	RF	0.930 (0.807–1.000)	0.830	0.974	0.222	0.841	0.667	0.902
	LightGBM	0.822 (0.578–1.000)	0.872	1.000	0.333	0.864	1.000	0.927
	XGBoost	0.899 (0.719–1.000)	0.851	0.974	0.333	0.860	0.750	0.914
	CatBoost	0.904 (0.781–0.986)	0.830	0.921	0.444	0.875	0.571	0.897
	AdaBoost	0.883 (0.686–1.000)	0.872	0.974	0.444	0.881	0.800	0.925
Strategy 4	NB	0.895 (0.696–1.000)	0.745	0.711	0.889	0.964	0.421	0.818
	LR	0.921 (0.798–0.994)	0.872	0.947	0.556	0.900	0.714	0.923
	SVM	0.915 (0.808–0.990)	0.894	0.974	0.556	0.902	0.833	0.937
	KNN	0.839 (0.673–0.971)	0.872	0.974	0.444	0.881	0.800	0.925
	ANN	0.816 (0.619–0.968)	0.894	0.974	0.556	0.902	0.833	0.937
	DT	0.775 (0.606–0.928)	0.766	0.868	0.333	0.846	0.375	0.857
	GBDT	0.939 (0.828–1.000)	0.936	0.974	0.778	0.949	0.875	0.961
	RF	0.936 (0.816–1.000)	0.872	0.974	0.444	0.881	0.800	0.925
	LightGBM	0.918 (0.750–1.000)	0.915	0.974	0.667	0.925	0.857	0.949
	XGBoost	0.953 (0.848–1.000)	0.915	0.974	0.667	0.925	0.857	0.949
	CatBoost	0.947 (0.856–1.000)	0.894	0.974	0.556	0.902	0.833	0.937
	AdaBoost	0.933 (0.804–1.000)	0.936	0.974	0.778	0.949	0.875	0.961

*Note:* Strategy 1, baseline clinicopathological features (age, maximum tumor diameter, ki67, EGFR, PD‐L1, histotype, grade, FIGO stage); Strategy 2, Strategy 1 + tumoral TGFBIp; Strategy 3, Strategy 1 + stromal TGFBIp; Strategy 4, Strategy 1 + both. For the test set, the AUC is given with the bootstrap 95% confidence interval (2000 resamples). Classification metrics use the 0.5 probability threshold.

Abbreviations: NPV, negative predictive value; PPV, positive predictive value.

For vital status prediction (Table 4), the SVM model with Strategy 4 achieved a test‐set AUC of 0.951 (95% CI 0.870–0.998), accuracy of 0.872, sensitivity of 0.920, specificity of 0.818, PPV of 0.852, NPV of 0.900, F1‐score of 0.885, AUPRC of 0.938, and Brier score of 0.071 (Figure S1). Training‐set performance was comparable (AUC 0.974; Table 3), indicating no substantial overfitting.

For recurrence prediction (Table 6), the RF model with Strategy 4 achieved a test‐set AUC of 0.936 (95% CI 0.816–1.000), accuracy of 0.872, sensitivity of 0.974, specificity of 0.444, PPV of 0.881, NPV of 0.800, F1‐score of 0.925, AUPRC of 0.984, and Brier score of 0.079 (Figure S1). The high sensitivity (0.974) and NPV (0.800) indicate that the model rarely missed recurrent cases, which is clinically desirable for a recurrence‐risk tool, although the modest specificity (0.444) reflects the strong class imbalance of the cohort (80.6% recurrence). A class‐weighted sensitivity analysis yielded consistent findings (data not shown).

### 3.4 Incremental Prognostic Value of TGFBIp

The incremental value of TGFBIp features was formally tested by paired bootstrap comparison of test‐set AUCs against the baseline strategy (Table S5, Figure S2). For vital status prediction with the SVM, adding both tumor and stromal TGFBIp (Strategy 4) significantly improved discrimination over baseline (ΔAUC +0.038, 95% CI +0.002 to +0.092). For recurrence prediction with the RF, Strategy 2 (adding tumoral TGFBIp) yielded a ΔAUC of +0.026 (95% CI 0.000 to +0.065), and Strategy 4 yielded +0.021 (95% CI −0.012 to +0.071). In the sensitivity analysis using LR as the reference algorithm, the incremental value of Strategy 4 was significant for both outcomes (vital status: ΔAUC +0.036, 95% CI 0.000 to +0.090; recurrence: ΔAUC +0.056, 95% CI +0.007 to +0.128). These results provide formal statistical support for the incremental prognostic value of TGFBIp, particularly tumoral TGFBIp.

### 3.5 SHAP Analysis of the Final Models

For vital status prediction (SVM, Strategy 4), maximum tumor diameter exhibited the highest mean absolute SHAP value, followed by Grade III, TGFBIp expression in tumor cells (ranked third), Stage IV, and TGFBIp expression in stroma (ranked fifth) (Figure 2A). Higher TGFBIp expression in both compartments was associated with increased predicted mortality risk. For recurrence prediction (RF, Strategy 4), Stage II and Stage III were the leading contributors, followed by TGFBIp expression in tumor cells (ranked third) and Grade III (Figure 2B); stromal TGFBIp did not rank among the top 10 features for recurrence. Thus, tumoral TGFBIp contributed to both prediction tasks, whereas the contribution of stromal TGFBIp was more specific to vital status prediction.

**FIGURE 2:**
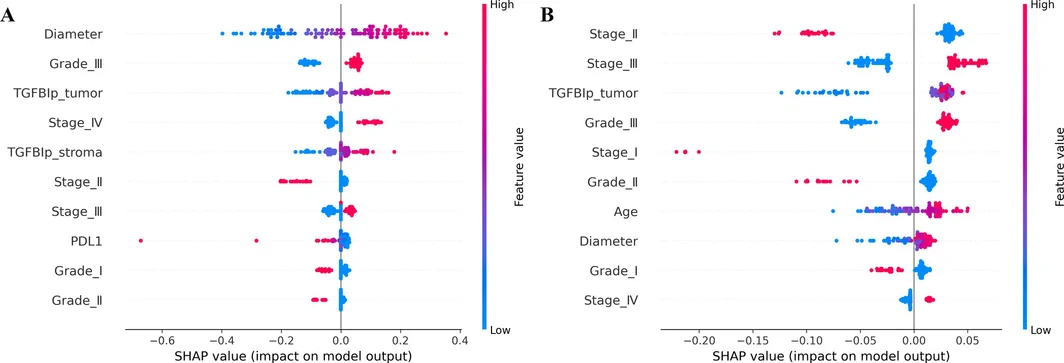
SHAP summary plots for the final models with Strategy 4. (A) SHAP summary plot for vital status prediction (support vector machine, KernelExplainer). (B) SHAP summary plot for recurrence prediction (random forest, TreeExplainer). Features are ranked by their mean absolute SHAP values, with the top 10 most influential features displayed. Each dot represents a single patient; the color gradient from blue to red represents increasing feature values. The horizontal position indicates the SHAP value, reflecting the contribution of each feature to the predicted risk. SHAP, SHapley Additive exPlanations.

### 3.6 Calibration Performance

Calibration was assessed using calibration curves, the calibration intercept and slope from logistic recalibration, and the Brier score (Figure 3, Table S6). For vital status, the SVM model showed acceptable calibration in the test set (intercept −0.24, slope 1.25, Brier score 0.071). For recurrence, the RF model showed a test‐set calibration intercept of −0.72, a slope of 1.62, and a Brier score of 0.079; the slope above 1 indicates that predicted probabilities were moderately under‐dispersed, a common pattern for tree‐ensemble models, and absolute probability estimates from the recurrence model should therefore be interpreted with caution.

**FIGURE 3:**
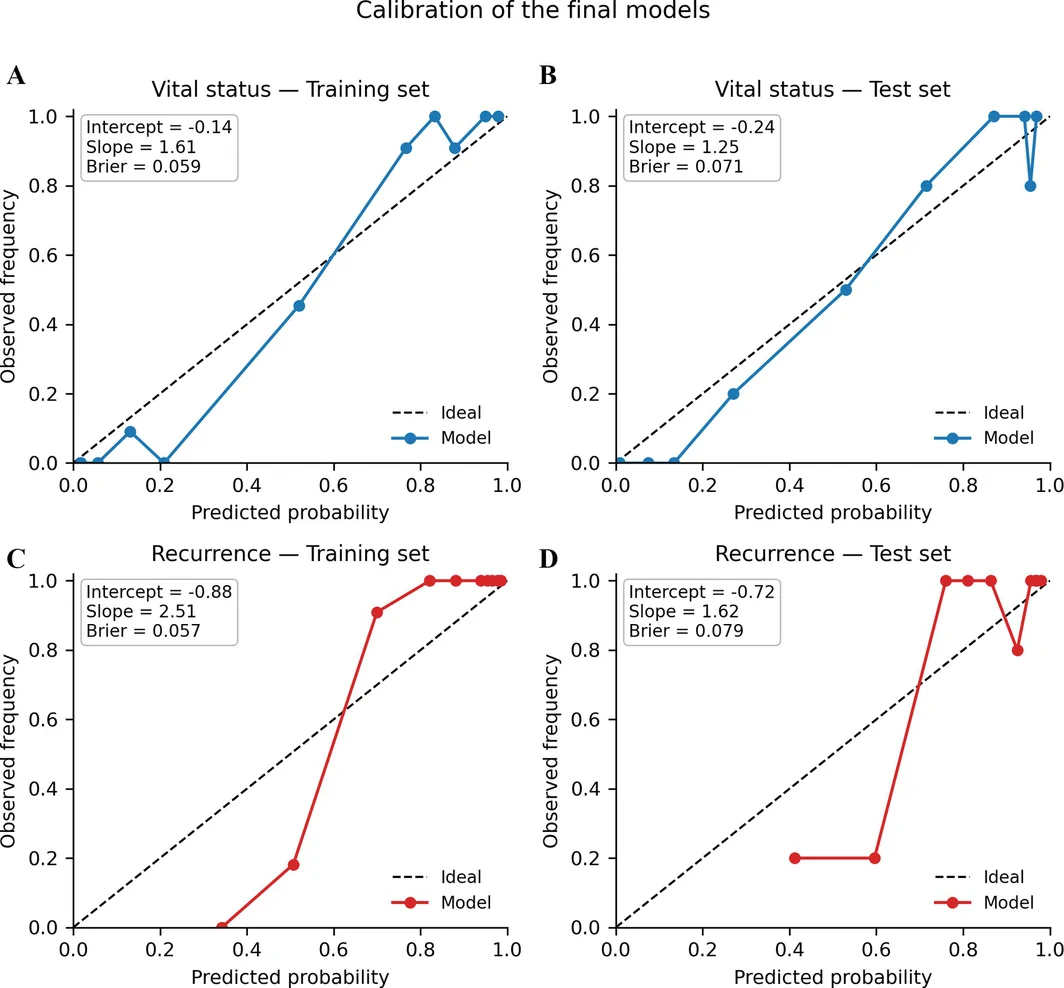
Calibration curves for the final models with Strategy 4. (A) Vital status prediction in the training set. (B) Vital status prediction in the test set. (C) Recurrence prediction in the training set. (D) Recurrence prediction in the test set. The diagonal dashed line indicates ideal calibration, where predicted probabilities perfectly match observed outcome frequencies. Calibration intercept, calibration slope (from logistic recalibration), and Brier score are annotated in each panel.

### 3.7 Decision Curve Analysis

DCA suggested potential clinical utility of both final models on the test set (Figure 4). For vital status prediction, the SVM model provided a net benefit exceeding both the “treat all” and “treat none” strategies across a broad range of threshold probabilities. For recurrence prediction, the RF model showed a higher net benefit than the reference strategies across the high‐threshold range, consistent with its high sensitivity. However, given the modest cohort size and the internal nature of the validation, these findings indicate potential rather than established clinical utility, and external validation is required before clinical application.

**FIGURE 4:**
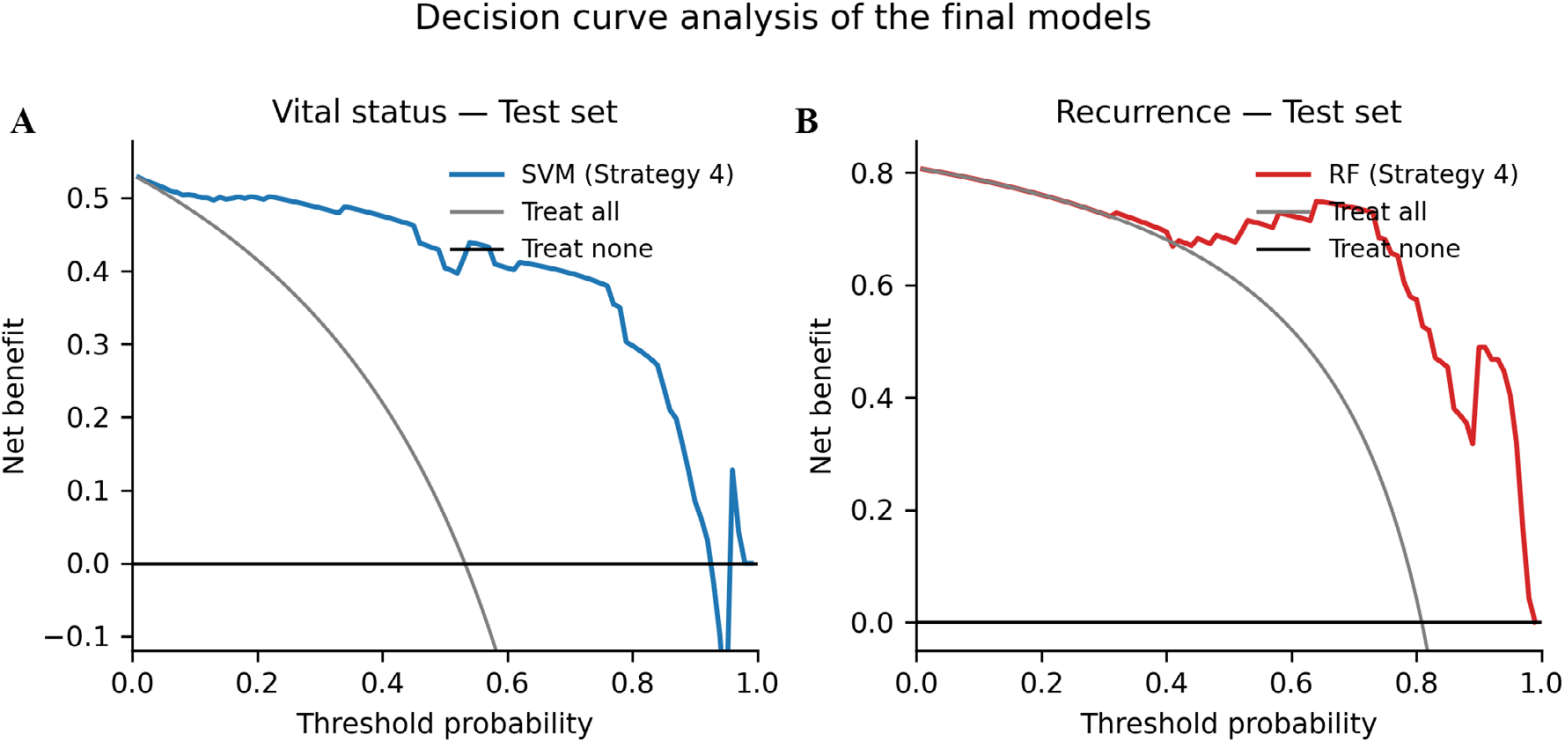
Decision curve analysis for the final models with Strategy 4 on the test set. (A) Vital status prediction (support vector machine). (B) Recurrence prediction (random forest). The y‐axis represents the net benefit, and the x‐axis represents the threshold probability. The grey line represents the “treat all” strategy, and the horizontal black line represents the “treat none” strategy. DCA, decision curve analysis.

Specifically, the vital status model could help clinicians decide how intensively each patient should be followed up. Because a false‐positive prediction mainly leads to additional follow‐up rather than serious harm, the model provides net benefit across a wide range of threshold probabilities. The recurrence model rarely misses recurrent cases (sensitivity 0.974, NPV 0.800), so patients predicted to be at low risk could safely remain under standard follow‐up. Its net benefit at higher threshold probabilities fits clinical scenarios in which clinicians would intervene only when the predicted risk is high, to avoid unnecessary procedures in patients who would not benefit.

## 4 Discussion

In this study, we developed and internally validated machine learning models to predict vital status and recurrence in EOC patients, with a focus on formally evaluating the incremental prognostic value of compartment‐specific TGFBIp expression. Using a leakage‐free analytical pipeline, with train‐test splitting performed before any preprocessing, hyperparameter tuning by cross‐validation on the training set, and final model selection by repeated cross‐validation on the training set only, SVM with Strategy 4 was selected for vital status prediction (test‐set AUC 0.951, 95% CI 0.870–0.998) and RF with Strategy 4 for recurrence prediction (test‐set AUC 0.936, 95% CI 0.816–1.000). Paired bootstrap testing demonstrated that adding TGFBIp features significantly improved vital status prediction, and SHAP analysis identified tumoral TGFBIp among the leading predictors for both outcomes. Decision curve analysis suggested potential clinical utility across a broad range of threshold probabilities; however, external validation is required before clinical application.

An important methodological aspect of this revision is the strict separation between model development and model evaluation. In small clinical cohorts, performing imputation, feature encoding, or model selection on the full dataset before splitting leads to information leakage and optimistically biased performance estimates [15, 16]. We therefore fitted all preprocessing steps on the training set only, tuned hyperparameters by 10‐fold cross‐validation within the training set, selected the final algorithm and feature strategy by repeated 5‐fold cross‐validation on the training set, and evaluated the untouched test set exactly once. Consistent with the expectation that leakage inflates performance, several flexible algorithms that appeared attractive under less stringent evaluation ranked below simpler regularized models under the present pipeline. This observation echoes recent methodological work showing that, with small sample sizes, simpler models with regularization often generalize more robustly than complex ensembles, which are more prone to capturing noise [15, 16].

The two outcomes favored different algorithms. For vital status, the SVM with radial basis function kernel combined high discrimination with the best out‐of‐fold calibration among the leading candidates, which motivated its selection. For recurrence, the RF achieved the highest repeated cross‐validation AUC and retained a high sensitivity (0.974) with an NPV of 0.800 on the test set, meaning that patients predicted as non‐recurrent were mostly recurrence‐free; this is a clinically desirable property for ruling out recurrence risk, although the modest specificity (0.444) must be acknowledged and largely reflects the high recurrence prevalence (80.6%) in this cohort, which is consistent with previous reports that over 80% of EOC patients experience relapse [17]. Class imbalance is known to distort the operating characteristics of machine learning classifiers, increasing false negative or false positive rates in the minority class [18, 19]; we addressed this by reporting AUPRC alongside AUC, by a class‐weighted sensitivity analysis, and by interpreting the classification metrics in light of the outcome prevalence.

A central finding of this study is the formal demonstration of the incremental prognostic value of TGFBIp. Adding TGFBIp features to the baseline clinicopathological model significantly improved vital status prediction (ΔAUC +0.038, 95% CI +0.002 to +0.092), and the direction and magnitude of the effect were consistent across algorithms and in the LR sensitivity analysis, in which the improvement was significant for both outcomes. These results are biologically plausible: a growing body of evidence has demonstrated that TGFBI expression is significantly associated with prognosis across multiple cancer types, with elevated TGFBIp levels generally correlating with poor overall and disease‐free survival [20]. In ovarian cancer, TGFBIp has been identified as an independent prognostic factor [8]. Stromal TGFBIp expression predicts poor prognosis in esophageal and gastric cancers [21, 22], and TGFBIp overexpression in cytoplasm or stroma correlates with adverse outcomes in colorectal and renal cancers [23, 24]. In line with this compartment‐specific biology, our SHAP analysis showed that tumoral TGFBIp contributed to both outcomes, whereas stromal TGFBIp contributed mainly to vital status prediction, suggesting that the two compartments carry related but not identical prognostic information.

Beyond TGFBIp, SHAP analysis identified maximum tumor diameter, histological grade, and FIGO stage among the most influential predictors. These findings are well supported by the literature. Maximum tumor diameter has been incorporated into nomogram models for ovarian cancer prognosis [25], and ovarian tumor diameter has been reported to be significantly associated with survival [26]. FIGO stage is recognized by the ESMO Clinical Practice Guidelines as a primary determinant of prognosis [27], and histological grade has been shown to predict survival outcomes in ovarian cancer [28, 29]. The convergence of our SHAP findings with these established prognostic factors supports the face validity of the models.

Discrimination and calibration are distinct yet complementary aspects of model performance [30, 31]. The vital status model showed acceptable calibration in the test set, whereas the recurrence model showed moderate miscalibration, with predicted probabilities that were under‐dispersed (calibration slope 1.62). Miscalibration of tree‐ensemble probability estimates is well documented, and recalibration is typically required when absolute risk estimates are needed [30]. We therefore recommend that the present models be used primarily for risk stratification rather than for absolute risk prediction, notably, relative risk stratification can remain valid even when absolute probability estimates are imperfectly calibrated [32].

Our binary classification framework differs conceptually from time‐to‐event prediction approaches. Time‐to‐event models exploit the exact timing and censoring of events and are generally preferred when complete event‐time data are available [12]. In the present commercial TMA cohort, follow‐up duration and outcome status were available, but follow‐up was heterogeneous (4–109 months) and the cohort size limited the number of events per variable for survival modeling. We therefore adopted binary endpoints at the end of follow‐up as a pragmatic framework for benchmarking the incremental value of TGFBIp across 12 classification algorithms, and we explicitly acknowledge that future work should re‐evaluate TGFBIp within time‐to‐event machine learning frameworks, such as survival RFs or deep survival models, in larger cohorts [12].

Recent years have witnessed growing interest in applying machine learning to ovarian cancer prognosis. A systematic review of 32 studies published between 2017 and 2023 found that RF, SVM, LR, and XGBoost were among the most commonly used algorithms for predicting overall and recurrence‐free survival in ovarian cancer [11, 12]. Most existing models have been built on large‐scale datasets such as SEER or TCGA [11] and have relied on high‐dimensional data sources, such as pathomics signatures [33], radiomics features [34], and multi‐omics profiles [35], to achieve predictive performance. While these studies demonstrate the feasibility of machine learning‐based prognostic modeling, the complexity of these data modalities limits their generalizability to routine clinical practice. In contrast, our framework uses only routinely available clinicopathological parameters and immunohistochemically detectable TGFBIp, a combination that is widely accessible in standard clinical settings, at a time when the management of ovarian cancer increasingly integrates surgery, chemotherapy, targeted agents, and emerging drug‐delivery strategies, including ligand‐targeted nanocarrier systems [27, 36, 37]. Furthermore, to our knowledge, this is the first study to systematically evaluate the incremental prognostic value of compartment‐specific TGFBIp expression in both tumor cells and tumor stroma within a machine learning framework, offering a novel perspective on integrating microenvironmental biomarkers into prognostic modeling.

Several limitations should be acknowledged. First, the sample size was modest (*n* = 155), which limits statistical power and the stability of performance estimates; small samples are a recognized challenge in machine learning studies of ovarian cancer [38]. Second, this was a retrospective, single‐cohort study based on a commercial tissue microarray, and the held‐out test set constitutes internal rather than external validation; external validation in independent, geographically distinct cohorts is essential before any clinical use. Third, the TMA dataset lacked detailed treatment information (chemotherapy regimens, surgical radicality, maintenance therapy) and molecular variables such as BRCA1/2 mutation status, all of which influence prognosis [11]. Fourth, binary endpoints at the end of follow‐up do not exploit event timing or censoring, and time‐to‐event re‐analysis is warranted. Fifth, TGFBIp expression was assessed by semi‐quantitative immunohistochemical scoring; although the blinded re‐scoring substudy showed moderate‐to‐good inter‐observer agreement (average‐measures ICC[2,2] ≈ 0.81 for both compartments), semi‐quantitative scoring remains inherently subject to observer variability, and future studies should incorporate digital pathology or automated quantification to further improve reproducibility. Finally, the recurrence model showed modest specificity and moderate miscalibration, reflecting the high recurrence prevalence and the modest number of non‐recurrent cases.

## 5 Conclusion

In conclusion, using a leakage‐free machine learning pipeline, we developed and internally validated interpretable models incorporating compartment‐specific TGFBIp expression for predicting vital status and recurrence in EOC. TGFBIp expression—particularly in tumor cells—provided incremental prognostic value beyond routine clinicopathological parameters, formally supported by paired bootstrap testing. Given the modest sample size, internal nature of the validation, and moderate calibration of the recurrence model, these models should be regarded as research tools; larger multicenter prospective studies with independent external validation and time‐to‐event re‐analysis are required before clinical implementation.

## Author Contributions

**Yi Guo:** funding acquisition, project administration, writing – review and editing. **Zhen Li:** formal analysis, methodology, software, validation. **Wenwei Song:** data curation, resources. **Lansheng Zhang:** funding acquisition, project administration, writing – review and editing.

## Funding

This work was supported by the National Natural Science Foundation of China (No. 82402722), the China Postdoctoral Science Foundation (No. 2023M732975), and the Key Research and Development Program of Xuzhou Science and Technology Bureau (No. KC23265).

## Ethics Statement

This study was approved by the Ethics Committee of the Second Affiliated Hospital of Xuzhou Medical University (approval No. [2024]030401), and was conducted in accordance with the principles of the Declaration of Helsinki. Ovarian tissues were procured from Shanghai Outdo Biotech Co. Ltd.

## Consent

Informed consent was obtained from all donors at the time of tissue collection, and the associated clinicopathological and follow‐up data were subsequently provided in a fully de‐identified form.

## Conflicts of Interest

The authors declare no conflicts of interest.

## Supporting information


**Figure S1:** Receiver operating characteristic and precision‐recall curves of the final models on the held‐out test set. (A) ROC for vital status. (B) Precision‐recall curve for vital status. (C) ROC for recurrence. (D) Precision‐recall curve for recurrence. AUC, area under the receiver operating characteristic curve; AUPRC, area under the precision‐recall curve; CI, confidence interval.
**Figure S2:** Test‐set receiver operating characteristic curves comparing the four feature inclusion strategies within the selected algorithm. (A) Vital status (support vector machine). (B) Recurrence (random forest). S1, baseline clinicopathological features; S2, S1 + tumoral TGFBIp; S3, S1 + stromal TGFBIp; S4, S1 + both.
**Figure S3:** Bland–Altman analysis of inter‐observer agreement for TGFBIp immunohistochemical scoring between the two pathologists (*n* = 155 cores, blinded independent re‐scoring). (A) Tumoral TGFBIp score. (B) Stromal TGFBIp score. The solid line indicates the mean difference between raters, and the dashed lines indicate the 95% limits of agreement (±1.96 SD).
**Table S1:** Hyperparameter search space and selected values for the final models.
**Table S2:** Variance inflation factors of the Strategy 4 feature set.
**Table S3:** Model selection for vital status: repeated stratified 5‐fold cross‐validation AUC on the training set (10 repeats, 50 estimates per model).
**Table S4:** Model selection for recurrence: repeated stratified 5‐fold cross‐validation AUC on the training set (10 repeats, 50 estimates per model).
**Table S5:** Incremental value of TGFBIp: paired bootstrap ΔAUC versus Strategy 1 on the held‐out test set.
**Table S6:** Calibration performance of the final models.

## Data Availability

The data that support the findings of this study are available from the corresponding author upon reasonable request.

## References

[1] A. Gonzalez‐Martin , P. Harter , A. Leary , et al., “Newly Diagnosed and Relapsed Epithelial Ovarian Cancer: ESMO Clinical Practice Guideline for Diagnosis, Treatment and Follow‐Up,” Annals of Oncology 34, no. 10 (2023): 833–848, 10.1016/j.annonc.2023.07.011. 37597580

[2] G. Caruso , S. J. Weroha , and W. Cliby , “Ovarian Cancer: A Review,” JAMA 334, no. 14 (2025): 1278–1291, 10.1001/jama.2025.9495. 40690248

[3] R. E. Eldesouki , O. Tarek , and H. Morsy , “Integrating Multi‐Omics and Clinical Features to Model Survival in Epithelial Ovarian Cancer Subtypes,” Scientific Reports 15, no. 1 (2025): 44221. 10.1038/s41598-025-29403-6 PMC12722313 41419500

[4] Y. Guo , W. Cui , Y. Pei , and D. Xu , “Platelets Promote Invasion and Induce Epithelial to Mesenchymal Transition in Ovarian Cancer Cells by TGF‐Beta Signaling Pathway,” Gynecologic Oncology 153, no. 3 (2019): 639–650. 10.1016/j.ygyno.2019.02.026 30928020

[5] H. Huang , Q. Tang , S. Li , Y. Qin , and G. Zhu , “TGFBI: A Novel Therapeutic Target for Cancer,” International Immunopharmacology 134 (2024): 112180, 10.1016/j.intimp.2024.112180. 38733822

[6] A. Corona and G. C. Blobe , “The Role of the Extracellular Matrix Protein TGFBI in Cancer,” Cellular Signalling 84 (2021): 110028, 10.1016/j.cellsig.2021.110028. 33940163

[7] M. P. Ween , M. K. Oehler , and C. Ricciardelli , “Transforming Growth Factor‐Beta‐Induced Protein (TGFBI)/(Betaig‐H3): A Matrix Protein With Dual Functions in Ovarian Cancer,” International Journal of Molecular Sciences 13, no. 8 (2012): 10461–10477. 10.3390/ijms130810461 PMC3431872 22949874

[8] H. Wang , Y. H. Xu , and Y. Guo , “Novel Prognostic Marker TGFBI Affects the Migration and Invasion Function of Ovarian Cancer Cells and Activates the Integrin alphavbeta3‐PI3K‐Akt Signaling Pathway,” Journal of Ovarian Research 17, no. 1 (2024): 50. 10.1186/s13048-024-01377-5 PMC10885438 38395907

[9] N. Nikolaou , D. Salazar , H. RaviPrakash , et al., “A Machine Learning Approach for Multimodal Data Fusion for Survival Prediction in Cancer Patients,” NPJ Precis Oncol 9, no. 1 (2025): 128, 10.1038/s41698-025-00917-6. PMC12053085 40325104

[10] L. A. Vale‐Silva and K. Rohr , “Long‐Term Cancer Survival Prediction Using Multimodal Deep Learning,” Scientific Reports 11, no. 1 (2021): 13505, 10.1038/s41598-021-92799-4. PMC8242026 34188098

[11] F. Asadi , M. Rahimi , N. Ramezanghorbani , and S. Almasi , “Comparing the Effectiveness of Artificial Intelligence Models in Predicting Ovarian Cancer Survival: A Systematic Review,” Cancer Reports 8, no. 3 (2025): e70138, 10.1002/cnr2.70138. PMC11920737 40103563

[12] J. Nakayama , M. McGaughey , G. Pindzola , et al., “Machine‐Learning Survival Models for Predicting Time to Recurrence in Epithelial Ovarian Cancer,” Gynecologic Oncology 204 (2026): 184–193, 10.1016/j.ygyno.2025.11.021. 41351944

[13] G. S. Collins , K. G. M. Moons , P. Dhiman , et al., “TRIPOD+AI Statement: Updated Guidance for Reporting Clinical Prediction Models That Use Regression or Machine Learning Methods,” BMJ 385 (2024): e078378, 10.1136/bmj-2023-078378. PMC11019967 38626948

[14] S. M. Lundberg and S. I. Lee , “A Unified Approach to Interpreting Model Predictions,” Advances in Neural Information Processing Systems 30 (2017): 4765–4774.

[15] M. Z. Naser , “A Review of Machine Learning With Small and Limited Data,” Journal of Big Data 13, no. 1 (2026): 18, 10.1186/s40537-025-01346-9.

[16] N. Pallikkavaliyaveetil and S. Chandrasekaran , “Small Data, Big Challenges: Machine‐ and Deep‐Learning Strategies for Data‐Limited Drug Discovery,” Advanced Drug Delivery Reviews 229 (2026): 115762, 10.1016/j.addr.2025.115762. PMC13094559 41421504

[17] H. Alizadeh , P. Akbarabadi , A. Dadfar , M. R. Tareh , and B. Soltani , “A Comprehensive Overview of Ovarian Cancer Stem Cells: Correlation With High Recurrence Rate, Underlying Mechanisms, and Therapeutic Opportunities,” Molecular Cancer 24, no. 1 (2025): 135, 10.1186/s12943-025-02345-3. PMC12057202 40329326

[18] M. Vasconcelos and L. Cavique , “Mitigating False Negatives in Imbalanced Datasets: An Ensemble Approach,” Expert Systems With Applications 262 (2025): 125674, 10.1016/j.eswa.2024.125674.

[19] I. Straw , G. Rees , and P. Nachev , “Sex‐Based Performance Disparities in Machine Learning Algorithms for Cardiac Disease Prediction: Exploratory Study,” Journal of Medical Internet Research 26 (2024): e46936, 10.2196/46936. PMC11384168 39186324

[20] Y. Chen , H. Zhao , Y. Feng , et al., “Pan‐Cancer Analysis of the Associations of TGFBI Expression With Prognosis and Immune Characteristics,” Frontiers in Molecular Biosciences 8 (2021): 745649, 10.3389/fmolb.2021.745649. PMC8521171 34671645

[21] D. Ozawa , T. Yokobori , M. Sohda , et al., “TGFBI Expression in Cancer Stromal Cells Is Associated With Poor Prognosis and Hematogenous Recurrence in Esophageal Squamous Cell Carcinoma,” Annals of Surgical Oncology 23, no. 1 (2016): 282–289, 10.1245/s10434-014-4259-4. 25448803

[22] M. Suzuki , T. Yokobori , N. Gombodorj , et al., “High Stromal Transforming Growth Factor Beta‐Induced Expression Is a Novel Marker of Progression and Poor Prognosis in Gastric Cancer,” Journal of Surgical Oncology 118, no. 6 (2018): 966–974. 10.1002/jso.25217 30260476

[23] L. G. Perez , J. Kempski , H. M. McGee , et al., “TGF‐Beta Signaling in Th17 Cells Promotes IL‐22 Production and Colitis‐Associated Colon Cancer,” Nature Communications 11, no. 1 (2020): 2608. 10.1038/s41467-020-16363-w PMC7248087 32451418

[24] S. Jorge , J. L. Capelo , W. LaFramboise , et al., “Absolute Quantitative Proteomics Using the Total Protein Approach to Identify Novel Clinical Immunohistochemical Markers in Renal Neoplasms,” BMC Medicine 19, no. 1 (2021): 196. 10.1186/s12916-021-02071-9 PMC8420025 34482820

[25] Y. Ren , Y. Jin , R. Xu , et al., “Development and Validation of a Nomogram for Prognostic Prediction in Ovarian Cancer Based on Tumor Proliferation and Immune‐Related Biomarkers in Chinese Patients,” International Journal of Women's Health 17 (2025): 2661–2670. 10.2147/IJWH.S517367 PMC12396520 40896421

[26] J. F. Stratton , P. Pharoah , J. A. Tidy , and M. E. Paterson , “An Analysis of Ovarian Tumor Diameter and Survival,” International Journal of Gynecological Cancer 10, no. 6 (2000): 449–451, 10.1046/j.1525-1438.2000.00070.x. 11240713

[27] A. Gonzalez‐Martin , J. A. Ledermann , and clinicalguidelines@esmo.org EGCEa , “ESMO Clinical Practice Guideline Express Update on the Management of Epithelial Ovarian Cancer,” ESMO Open 11, no. 2 (2026): 106032. 10.1016/j.esmoop.2025.106032 PMC12947637 41741111

[28] S. Y. Ryu , S. I. Park , B. H. Nam , et al., “Prognostic Significance of Histological Grade in Clear‐Cell Carcinoma of the Ovary: A Retrospective Study of Korean Gynecologic Oncology Group,” Annals of Oncology 20, no. 6 (2009): 1032–1036, 10.1093/annonc/mdn764. 19193704

[29] H. Tanimoto , K. Shigemasa , X. Tian , et al., “Transmembrane Serine Protease TADG‐15 (ST14/Matriptase/MT‐SP1): Expression and Prognostic Value in Ovarian Cancer,” British Journal of Cancer 92, no. 2 (2005): 278–283, 10.1038/sj.bjc.6602320. PMC2361855 15611789

[30] B. Van Calster , D. J. McLernon , M. van Smeden , L. Wynants , and E. W. Steyerberg , “Calibration: The Achilles Heel of Predictive Analytics,” BMC Medicine 17, no. 1 (2019): 230, 10.1186/s12916-019-1466-7. PMC6912996 31842878

[31] O. Efthimiou , M. Seo , K. Chalkou , T. Debray , M. Egger , and G. Salanti , “Developing Clinical Prediction Models: A Step‐By‐Step Guide,” BMJ 386 (2024): e078276, 10.1136/bmj-2023-078276. PMC11369751 39227063

[32] N. R. Cook , “Use and Misuse of the Receiver Operating Characteristic Curve in Risk Prediction,” Circulation 115, no. 7 (2007): 928–935, 10.1161/CIRCULATIONAHA.106.672402. 17309939

[33] H. Yin , Q. Liu , S. Feng , et al., “Development of the Model for Predicting the Efficacy of Bevacizumab and Prognosis in Ovarian Cancer Using Deep Learning‐Based Pathomics Signatures,” Chinese Medical Journal (English) 139, no. 5 (2026): 773–775, 10.1097/CM9.0000000000003823. PMC12959794 41424025

[34] S. Xu , C. Zhu , M. Wu , et al., “Artificial Intelligence Algorithm for Preoperative Prediction of FIGO Stage in Ovarian Cancer Based on Clinical Features Integrated 18F‐FDG PET/CT Metabolic and Radiomics Features,” Journal of Cancer Research and Clinical Oncology 151, no. 2 (2025): 87, 10.1007/s00432-025-06134-9. PMC11842498 39976736

[35] X. Qi , C. Jiang , N. Wang , et al., “Identification of Parthanatos‐Related Molecular Subtypes and Development of Prognostic Risk Models in Ovarian Cancer Based on Multi‐Omics Analysis,” Journal of Ovarian Research 18, no. 1 (2025): 137, 10.1186/s13048-025-01726-y. PMC12217911 40605104

[36] S. Bose , S. Sharma , A. Kumar , Y. Mishra , and V. Mishra , “Ovarian Cancer and Its Management Through Advanced Drug Delivery System,” Medical Oncology 42, no. 3 (2025): 76, 10.1007/s12032-025-02621-8. 39960609

[37] A. Chattaraj , Y. Mishra , A. A. A. Aljabali , and V. Mishra , “Development and Evaluation of Folic Acid Conjugated Curcumin‐Loaded Functionalized Multiwalled Carbon Nanotubes for Enhanced Efficacy in Ovarian Cancer Treatment,” Carbon Trends 19 (2025): 100464, 10.1016/j.cartre.2025.100464.

[38] P. Ordas , J. Crossa , and L. Chiva , “Artificial Intelligence for Single‐Omics in Ovarian Cancer: A Methodological Review,” International Journal of Gynecological Cancer 36, no. 4 (2026): 104452, 10.1016/j.ijgc.2025.104452. 41617590

