# Supplementary material for: Development and Validation of Machine Learning Models Incorporating TGFBIp Expression for Prognosis Prediction in Epithelial Ovarian Cancer
Source: Cancer Rep (Hoboken). 2026 Nov 10;9(11):e70745. doi: 10.1002/cnr2.70745 (PMC13749431; doi:10.1002/cnr2.70745)
Supplement: Supplementary file 1 [file CNR2-9-e70745-s001.docx]

**Supplementary Materials**

Figure S1. Receiver operating characteristic and precision-recall curves of the final models on the held-out test set. (A) ROC for vital status. (B) Precision-recall curve for vital status. (C) ROC for recurrence. (D) Precision-recall curve for recurrence. Abbreviations: AUC, area under the receiver operating characteristic curve; AUPRC, area under the precision-recall curve; CI, confidence interval.

Figure S2. Test-set receiver operating characteristic curves comparing the four feature inclusion strategies within the selected algorithm. (A) Vital status (support vector machine). (B) Recurrence (random forest). S1, baseline clinicopathological features; S2, S1 + tumoral TGFBIp; S3, S1 + stromal TGFBIp; S4, S1 + both.

Figure S3. Bland-Altman analysis of inter-observer agreement for TGFBIp immunohistochemical scoring between the two pathologists (n = 155 cores, blinded independent re-scoring). (A) Tumoral TGFBIp score. (B) Stromal TGFBIp score. The solid line indicates the mean difference between raters, and the dashed lines indicate the 95% limits of agreement (±1.96 SD).

Table S1. Hyperparameter search space and selected values for the final models.

| Algorithm | Search space (10-fold CV on the training set) | Selected - vital status (Strategy 4) | Selected - recurrence (Strategy 4) |
| --- | --- | --- | --- |
| NB | var_smoothing: 1e-12–1e-6 (7 log-spaced) | {"var_smoothing": 1e-12} | {"var_smoothing": 1e-12} |
| LR | C: 0.01–100 (5 log-spaced); L2; lbfgs | {"C": 100, "penalty": "l2", "solver": "lbfgs"} | {"C": 1, "penalty": "l2", "solver": "lbfgs"} |
| SVM | C: 0.1/1/10; gamma: scale/0.01/0.1; RBF kernel | {"C": 10, "gamma": 0.01, "kernel": "rbf"} | {"C": 10, "gamma": 0.01, "kernel": "rbf"} |
| KNN | n_neighbors: 3/5/7/11/15; weights: uniform/distance | {"n_neighbors": 5, "weights": "distance"} | {"n_neighbors": 7, "weights": "distance"} |
| ANN | hidden layers: (16,)/(32,)/(16,8); alpha: 1e-4/1e-3/1e-2 | {"alpha": 0.0001, "hidden_layer_sizes": "(32,)"} | {"alpha": 0.001, "hidden_layer_sizes": "(16,)"} |
| DT | max_depth: 2/3/4/5/None; min_samples_leaf: 1/5/10 | {"max_depth": 4, "min_samples_leaf": 5} | {"max_depth": 5, "min_samples_leaf": 1} |
| GBDT | n_estimators: 100/200; learning_rate: 0.05/0.1; max_depth: 2/3 | {"learning_rate": 0.1, "max_depth": 2, "n_estimators": 200} | {"learning_rate": 0.05, "max_depth": 2, "n_estimators": 100} |
| RF | n_estimators: 200/500; max_depth: 3/5/None; min_samples_leaf: 1/5 | {"max_depth": 3, "min_samples_leaf": 1, "n_estimators": 500} | {"max_depth": 3, "min_samples_leaf": 1, "n_estimators": 200} |
| LightGBM | n_estimators: 100/200; learning_rate: 0.05/0.1; num_leaves: 15/31; max_depth: 3/-1 | {"learning_rate": 0.05, "max_depth": 3, "n_estimators": 100, "num_leaves": 15} | {"learning_rate": 0.05, "max_depth": 3, "n_estimators": 100, "num_leaves": 15} |
| XGBoost | n_estimators: 100/200; learning_rate: 0.05/0.1; max_depth: 2/3/4 | {"learning_rate": 0.05, "max_depth": 2, "n_estimators": 100} | {"learning_rate": 0.05, "max_depth": 4, "n_estimators": 100} |
| CatBoost | iterations: 100/200; learning_rate: 0.05/0.1; depth: 3/4/6 | {"depth": 4, "iterations": 100, "learning_rate": 0.05} | {"depth": 4, "iterations": 200, "learning_rate": 0.05} |
| AdaBoost | n_estimators: 50/100/200; learning_rate: 0.05/0.1/0.5/1.0; base depth: 1/2 | {"estimator": "DecisionTreeClassifier(max_depth=1)", "learning_rate": 0.1, "n_estimators": 200} | {"estimator": "DecisionTreeClassifier(max_depth=1)", "learning_rate": 0.5, "n_estimators": 50} |

*Hyperparameters were tuned by grid search with stratified 10-fold cross-validation on the training set only. Selected values are shown for the final models (SVM for vital status; RF for recurrence), both under Strategy 4.*

Table S2. Variance inflation factors of the Strategy 4 feature set.

| Feature (one-hot encoded) | VIF |
| --- | --- |
| Histotype_serous carcinoma | 11.74 |
| Stage_Ⅲ | 9.84 |
| Stage_Ⅳ | 6.36 |
| Grade_Ⅲ | 6.20 |
| Stage_Ⅱ | 4.23 |
| Histotype_mucinous carcinoma | 3.65 |
| Histotype_endometrioid carcinoma | 2.71 |
| Grade_Ⅱ | 1.75 |
| TGFBIp_tumor | 1.53 |
| TGFBIp_stroma | 1.32 |
| Diameter | 1.25 |
| ki67 | 1.20 |
| EGFR | 1.15 |
| PDL1 | 1.15 |
| Age | 1.14 |

*VIFs were computed on the one-hot encoded Strategy 4 design matrix of the training set. All values were below 12, and all but Histotype_serous carcinoma (11.74) were below 10.*

Table S3. Model selection for vital status: repeated stratified 5-fold cross-validation AUC on the training set (10 repeats, 50 estimates per model).

| Rank | Feature strategy | Algorithm | AUC (mean ± SD) |
| --- | --- | --- | --- |
| 1 | Strategy 4 | SVM | 0.9375 ± 0.0413 |
| 2 | Strategy 4 | XGBoost | 0.9360 ± 0.0474 |
| 3 | Strategy 4 | ANN | 0.9358 ± 0.0458 |
| 4 | Strategy 4 | RF | 0.9352 ± 0.0493 |
| 5 | Strategy 2 | RF | 0.9351 ± 0.0488 |
| 6 | Strategy 3 | ANN | 0.9347 ± 0.0440 |
| 7 | Strategy 4 | CatBoost | 0.9320 ± 0.0527 |
| 8 | Strategy 2 | XGBoost | 0.9307 ± 0.0532 |
| 9 | Strategy 2 | SVM | 0.9305 ± 0.0450 |
| 10 | Strategy 1 | CatBoost | 0.9305 ± 0.0503 |
| 11 | Strategy 4 | AdaBoost | 0.9289 ± 0.0569 |
| 12 | Strategy 1 | AdaBoost | 0.9275 ± 0.0559 |
| 13 | Strategy 3 | CatBoost | 0.9272 ± 0.0551 |
| 14 | Strategy 1 | ANN | 0.9270 ± 0.0610 |
| 15 | Strategy 2 | ANN | 0.9268 ± 0.0560 |
| 16 | Strategy 2 | CatBoost | 0.9265 ± 0.0514 |
| 17 | Strategy 1 | RF | 0.9255 ± 0.0558 |
| 18 | Strategy 3 | AdaBoost | 0.9242 ± 0.0539 |
| 19 | Strategy 3 | RF | 0.9236 ± 0.0549 |
| 20 | Strategy 1 | XGBoost | 0.9213 ± 0.0560 |
| 21 | Strategy 3 | XGBoost | 0.9207 ± 0.0557 |
| 22 | Strategy 2 | AdaBoost | 0.9196 ± 0.0543 |
| 23 | Strategy 2 | KNN | 0.9160 ± 0.0535 |
| 24 | Strategy 3 | LR | 0.9145 ± 0.0617 |
| 25 | Strategy 1 | GBDT | 0.9139 ± 0.0587 |
| 26 | Strategy 1 | SVM | 0.9136 ± 0.0525 |
| 27 | Strategy 4 | GBDT | 0.9110 ± 0.0600 |
| 28 | Strategy 2 | GBDT | 0.9096 ± 0.0620 |
| 29 | Strategy 4 | NB | 0.9054 ± 0.0652 |
| 30 | Strategy 1 | LR | 0.9053 ± 0.0720 |
| 31 | Strategy 3 | GBDT | 0.9052 ± 0.0655 |
| 32 | Strategy 2 | NB | 0.9051 ± 0.0660 |
| 33 | Strategy 4 | LightGBM | 0.9051 ± 0.0623 |
| 34 | Strategy 4 | LR | 0.9046 ± 0.0727 |
| 35 | Strategy 2 | LR | 0.9046 ± 0.0702 |
| 36 | Strategy 3 | NB | 0.9019 ± 0.0682 |
| 37 | Strategy 3 | KNN | 0.9012 ± 0.0568 |
| 38 | Strategy 1 | NB | 0.9010 ± 0.0695 |
| 39 | Strategy 1 | LightGBM | 0.9000 ± 0.0655 |
| 40 | Strategy 1 | KNN | 0.8962 ± 0.0611 |
| 41 | Strategy 3 | LightGBM | 0.8961 ± 0.0647 |
| 42 | Strategy 2 | LightGBM | 0.8941 ± 0.0644 |
| 43 | Strategy 4 | KNN | 0.8937 ± 0.0610 |
| 44 | Strategy 1 | DT | 0.8814 ± 0.0707 |
| 45 | Strategy 4 | DT | 0.8745 ± 0.0724 |
| 46 | Strategy 3 | DT | 0.8688 ± 0.0809 |
| 47 | Strategy 2 | DT | 0.8653 ± 0.0778 |
| 48 | Strategy 3 | SVM | 0.4281 ± 0.4060 |

*The algorithm and feature strategy were selected on the training set only: the algorithm with the highest mean repeated-CV AUC was chosen first, and the feature strategy was then compared within the selected algorithm using the one-standard-error rule with out-of-fold calibration slope as the tie-breaker.*

Table S4. Model selection for recurrence: repeated stratified 5-fold cross-validation AUC on the training set (10 repeats, 50 estimates per model).

| Rank | Feature strategy | Algorithm | AUC (mean ± SD) |
| --- | --- | --- | --- |
| 1 | Strategy 4 | RF | 0.9236 ± 0.0618 |
| 2 | Strategy 2 | RF | 0.9212 ± 0.0633 |
| 3 | Strategy 1 | CatBoost | 0.9170 ± 0.0692 |
| 4 | Strategy 3 | RF | 0.9159 ± 0.0683 |
| 5 | Strategy 3 | CatBoost | 0.9147 ± 0.0616 |
| 6 | Strategy 4 | CatBoost | 0.9141 ± 0.0638 |
| 7 | Strategy 1 | RF | 0.9132 ± 0.0650 |
| 8 | Strategy 1 | GBDT | 0.9120 ± 0.0634 |
| 9 | Strategy 2 | CatBoost | 0.9096 ± 0.0666 |
| 10 | Strategy 4 | GBDT | 0.9092 ± 0.0761 |
| 11 | Strategy 1 | ANN | 0.9089 ± 0.0726 |
| 12 | Strategy 2 | ANN | 0.9051 ± 0.0765 |
| 13 | Strategy 3 | ANN | 0.9046 ± 0.0788 |
| 14 | Strategy 2 | SVM | 0.9046 ± 0.0747 |
| 15 | Strategy 2 | AdaBoost | 0.9041 ± 0.0757 |
| 16 | Strategy 1 | SVM | 0.9040 ± 0.0697 |
| 17 | Strategy 3 | GBDT | 0.9021 ± 0.0659 |
| 18 | Strategy 4 | AdaBoost | 0.9015 ± 0.0794 |
| 19 | Strategy 4 | SVM | 0.9012 ± 0.0688 |
| 20 | Strategy 1 | AdaBoost | 0.9003 ± 0.0773 |
| 21 | Strategy 2 | LightGBM | 0.8993 ± 0.0765 |
| 22 | Strategy 2 | GBDT | 0.8987 ± 0.0765 |
| 23 | Strategy 3 | SVM | 0.8987 ± 0.0839 |
| 24 | Strategy 1 | LR | 0.8985 ± 0.0730 |
| 25 | Strategy 4 | ANN | 0.8985 ± 0.0803 |
| 26 | Strategy 1 | DT | 0.8948 ± 0.0834 |
| 27 | Strategy 3 | DT | 0.8936 ± 0.0839 |
| 28 | Strategy 4 | LR | 0.8926 ± 0.0818 |
| 29 | Strategy 3 | AdaBoost | 0.8925 ± 0.0766 |
| 30 | Strategy 4 | LightGBM | 0.8923 ± 0.0744 |
| 31 | Strategy 2 | LR | 0.8919 ± 0.0806 |
| 32 | Strategy 3 | LR | 0.8915 ± 0.0782 |
| 33 | Strategy 4 | XGBoost | 0.8897 ± 0.0791 |
| 34 | Strategy 1 | KNN | 0.8860 ± 0.0756 |
| 35 | Strategy 2 | XGBoost | 0.8845 ± 0.0829 |
| 36 | Strategy 3 | XGBoost | 0.8843 ± 0.0881 |
| 37 | Strategy 1 | XGBoost | 0.8757 ± 0.0909 |
| 38 | Strategy 3 | NB | 0.8753 ± 0.1287 |
| 39 | Strategy 1 | NB | 0.8750 ± 0.1295 |
| 40 | Strategy 2 | DT | 0.8720 ± 0.1119 |
| 41 | Strategy 4 | NB | 0.8718 ± 0.1297 |
| 42 | Strategy 2 | NB | 0.8703 ± 0.1306 |
| 43 | Strategy 3 | LightGBM | 0.8695 ± 0.0891 |
| 44 | Strategy 3 | KNN | 0.8657 ± 0.0921 |
| 45 | Strategy 2 | KNN | 0.8647 ± 0.1002 |
| 46 | Strategy 1 | LightGBM | 0.8467 ± 0.1089 |
| 47 | Strategy 4 | KNN | 0.8462 ± 0.0978 |
| 48 | Strategy 4 | DT | 0.7814 ± 0.1166 |

*The algorithm and feature strategy were selected on the training set only: the algorithm with the highest mean repeated-CV AUC was chosen first, and the feature strategy was then compared within the selected algorithm using the one-standard-error rule with out-of-fold calibration slope as the tie-breaker.*

Table S5. Incremental value of TGFBIp: paired bootstrap ΔAUC versus Strategy 1 on the held-out test set.

| Model | Comparison | ΔAUC (95% CI) |
| --- | --- | --- |
| Vital status - SVM | Strategy 2 vs Strategy 1 | +0.015 (-0.035 to +0.082) |
| Vital status - SVM | Strategy 3 vs Strategy 1 | -0.009 (-0.065 to +0.049) |
| Vital status - SVM | Strategy 4 vs Strategy 1 | +0.038 (+0.002 to +0.092) |
| Recurrence - RF | Strategy 2 vs Strategy 1 | +0.026 (+0.000 to +0.065) |
| Recurrence - RF | Strategy 3 vs Strategy 1 | +0.015 (-0.018 to +0.057) |
| Recurrence - RF | Strategy 4 vs Strategy 1 | +0.021 (-0.012 to +0.071) |
| Vital status - LR (sensitivity analysis) | Strategy 2 vs Strategy 1 | +0.035 (+0.002 to +0.082) |
| Vital status - LR (sensitivity analysis) | Strategy 3 vs Strategy 1 | +0.024 (-0.011 to +0.073) |
| Vital status -LR (sensitivity analysis) | Strategy 4 vs Strategy 1 | +0.036 (+0.000 to +0.090) |
| Recurrence - LR (sensitivity analysis) | Strategy 2 vs Strategy 1 | +0.026 (-0.035 to +0.098) |
| Recurrence - LR (sensitivity analysis) | Strategy 3 vs Strategy 1 | +0.000 (+0.000 to +0.000) |
| Recurrence - LR (sensitivity analysis) | Strategy 4 vs Strategy 1 | +0.056 (+0.007 to +0.128) |

*ΔAUC is the difference in test-set AUC relative to Strategy 1 (baseline features only), with 95% confidence intervals from 2,000 paired bootstrap resamples. A confidence interval excluding zero indicates statistically significant incremental value. LR, logistic regression.*

Table S6. Calibration performance of the final models.

| Model | Set | Calibration intercept | Calibration slope | Brier score |
| --- | --- | --- | --- | --- |
| Vital status — SVM (Strategy 4) | Training | -0.14 | 1.61 | 0.059 |
| Vital status — SVM (Strategy 4) | Test | -0.24 | 1.25 | 0.071 |
| Recurrence — RF (Strategy 4) | Training | -0.88 | 2.51 | 0.057 |
| Recurrence — RF (Strategy 4) | Test | -0.72 | 1.62 | 0.079 |

*Calibration intercept and slope were estimated by logistic recalibration of the model-predicted probabilities against the observed outcomes.*
